# Author Correction: Adipose HuR protects against diet-induced obesity and insulin resistance

**DOI:** 10.1038/s41467-026-79109-0

**Published:** 2026-11-04

**Authors:** Jingyuan Li, Li Gong, Shaozhuang Liu, Yujie Zhang, Chunmei Zhang, Mi Tian, Huixia Lu, Peili Bu, Jianmin Yang, Changhan Ouyang, Xiuxin Jiang, Jiliang Wu, Yun Zhang, Qing Min, Cheng Zhang, Wencheng Zhang

**Affiliations:** 1 The Key Laboratory of Cardiovascular Remodeling and Function Research, Chinese Ministry of Education, Chinese National Health Commission and Chinese Academy of Medical Sciences, The State and Shandong Province Joint Key Laboratory of Translational Cardiovascular Medicine, Department of Cardiology, Qilu Hospital of Shandong University, Jinan, 250012 China; 2 Department of Traditional Chinese Integrated Western Medicine, Qilu Hospital of Shandong University, Jinan, 250012 China; 3 Department of General Surgery, Qilu Hospital of Shandong University, Jinan, 250012 China; 4 School of Pharmacy, Hubei University of Science and Technology, Xianning, 437100 Hubei China

Correction to: *Nature Communications* https://doi.org/10.1038/s41467-019-10348-0, published online 30 May 2019

In the version of this article initially published, in the Fig. 3g and Supplementary Fig. 2d Source Data and associated figure plots, the data blocks F/N (Fig. 3g) and block D (Supplementary Fig. 2d) were inadvertently copied and pasted into columns G/O (Fig. 3g) and block E (Supplementary Fig. 2d). Due to the age of the article, the Source Data and figure cannot be updated directly. The revised files are available as Supplementary Information accompanying this amendment. This notice serves to correct the article.

## Supplementary information

Corrected Fig. 3, Supplementary Fig. 2

Corrected Source Data

