# Supplementary material for: Adipose HuR protects against diet-induced obesity and insulin resistance
Source: Nat Commun. 2019 May 30;10:2375. doi: 10.1038/s41467-019-10348-0 (PMC6542850; doi:10.1038/s41467-019-10348-0)
Supplement: Supplementary file 1 — Supplementary Information [file 41467_2019_10348_MOESM1_ESM.pdf]

SUPPLEMENTARY INFORMATION

Adipose HuR protects against diet-induced obesity and insulin resistance

Li et al.

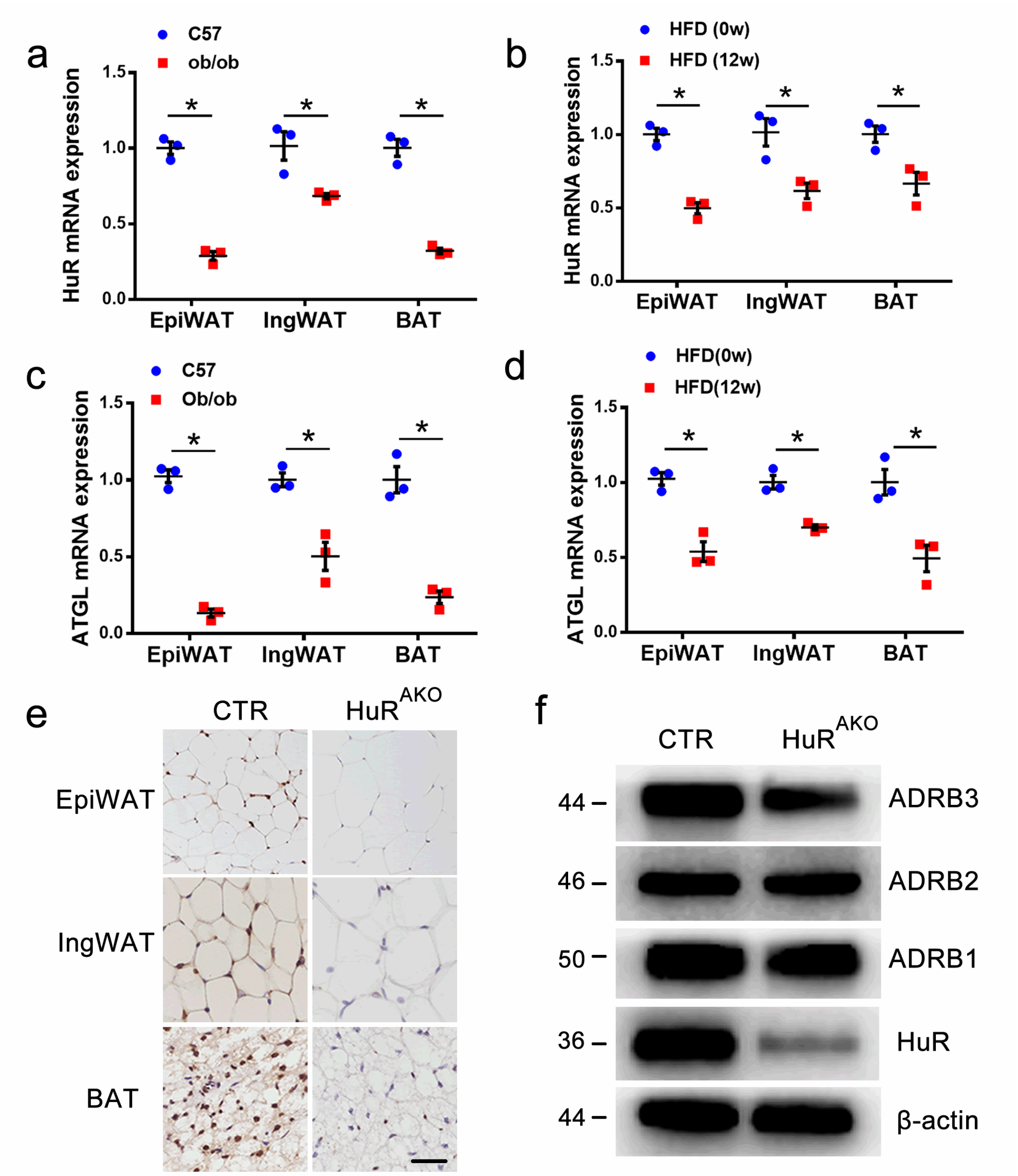

Supplementary Fig. 1 Identification of HuR and ATGL expression in mice. **a** qPCR analysis of *HuR* mRNA levels in adipose tissue (epiWAT, ingWAT and BAT) of 20-week-old C57BL/6J and ob/ob mice and quantification (n=3), *comparison of ob/ob *vs* C57. **b** Eight-week-old male C57BL/6J mice were fed with a HFD for an additional 12 weeks; western blot analysis of HuR and β-actin in adipose tissue and quantification (n=3), *comparison of HFD (12w) *vs* HFD (0w). **c-d** qPCR analysis of *ATGL* mRNA levels in adipose tissues. **e** Immunohistochemical staining of HuR in adipose tissues. Scale bar: 50 μm. **f** Western blot analysis of beta receptors (ADRB1, ADRB2, ADRB3) in CTR and HuR^{AKO} adipose tissues. Data are represented as mean ± SEM. Significance was determined by Student's t-test analysis, *P<0.05. Source data are provided as a Source Data file.

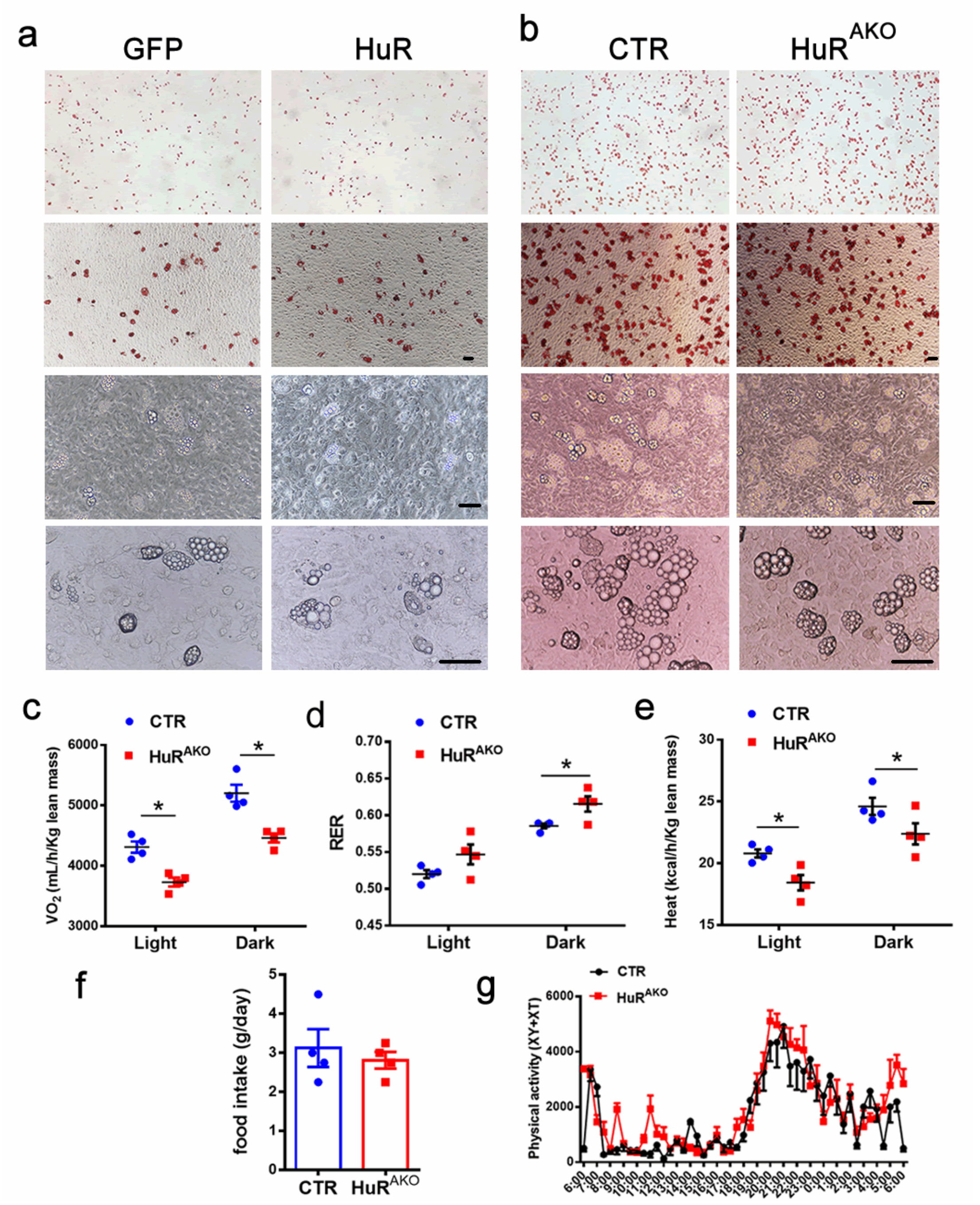

Supplementary Fig. 2 *HuR* doesn't affect adipose differentiation. **a-b** Representative images of differentiated lipid droplets. **a** 3T3-L1 cells were infected with adenovirus expressing GFP or HuR for 48 hours and then were induced to differentiation. Scale bar: 50 μm. **b** SVFs isolated from control and HuR^{AKO} mice were differentiated to adipocytes. Scale bar: 50 μm. **c-f** Quantitative analysis of metabolism studies from control and HuR^{AKO} mice fed an HFD: oxygen (O_2) consumption (**c**), respiratory exchange ratio (RER) (**d**), heat production (**e**) and food intake (**f**) (n=4), *comparison of HuR^{AKO} *vs* control. **g** Physical activity analysis of control and HuR^{AKO} mice fed an HFD (n=4). Data are represented as mean ± SEM. Significance was determined by Student's t-test analysis, *$P<0.05$. Source data are provided as a Source Data file.

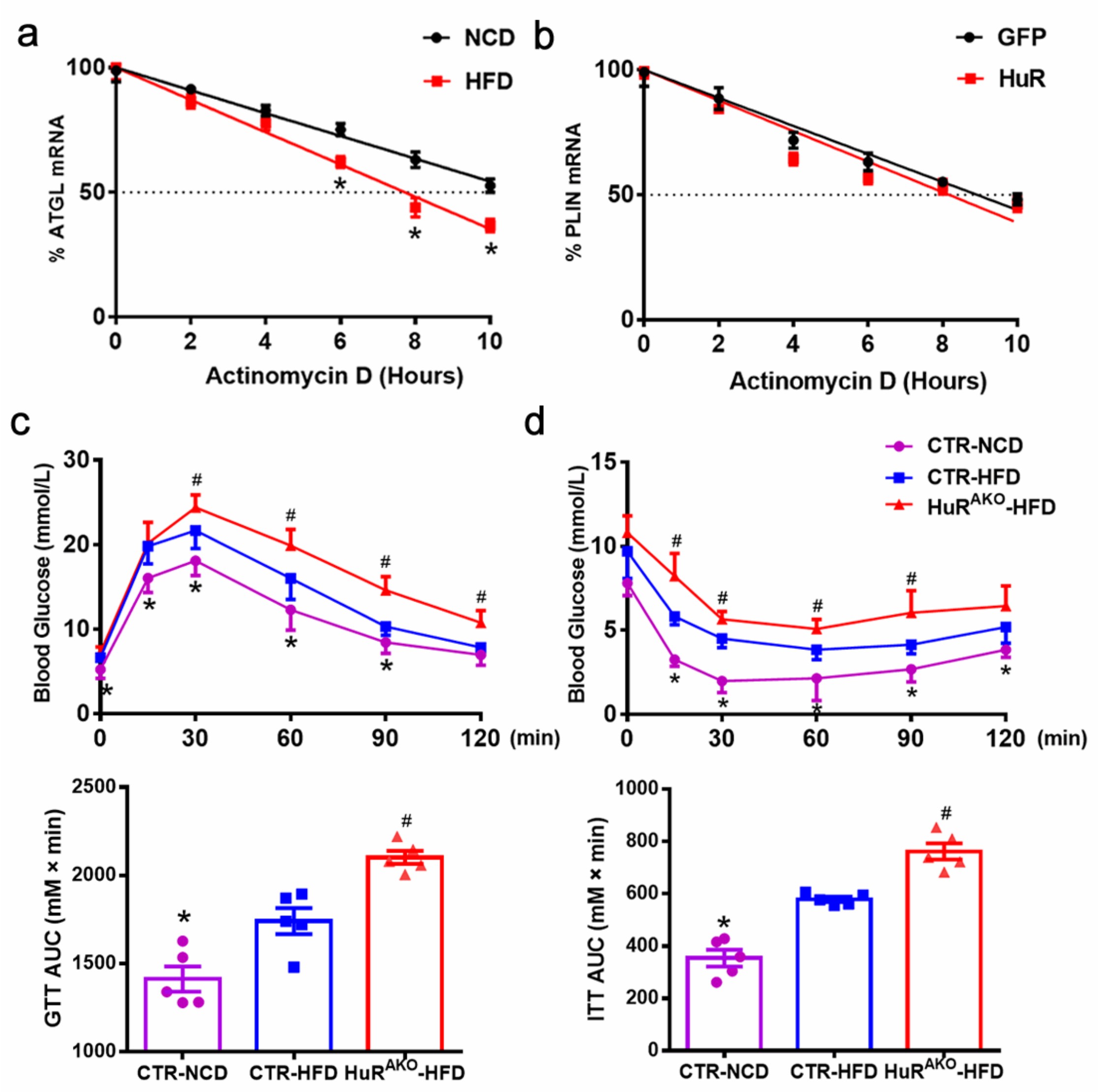

Supplementary Fig. 3 *HuR* deletion exacerbates insulin resistance. **a** SVFs isolated from control and HuR^{AKO} mice were differentiated to adipocytes and stimulated with 10 μg mL^{-1} actinomycin D. *ATGL* mRNA level was determined by qPCR (n=3). *comparison of HFD *vs* NCD. **b** Differentiated 3T3-L1 adipocytes were infected with adenovirus expressing GFP or *HuR* for 48 hours and stimulated with 10 μg mL^{-1} actinomycin D. *PLIN* mRNA level was determined by qPCR (n=3). **c-d** Glucose tolerance test (**c**) and insulin tolerance test (**d**) in NCD and HFD-fed control and HFD-fed HuR^{AKO} mice (n=5). Lower panel, area under curve. *comparison of CTR-NCD *vs* CTR-HFD, #comparison of HuR^{AKO}-HFD *vs* CTR-HFD. Data are represented as mean ± SEM. Significance was determined by Student's t-test analysis (**a-b**) and one-way analysis of variance (ANOVA) (**c-d**), *P<0.05, # P<0.05. Source data are provided as a Source Data file.

Genes	Forward	Reverse
HuR	ACACTGAACGGCTTGAGACT	CCTCTGGACAAACCTGTGGTC
C/EBPα	CTAGGAGATTCCGGTGTGGC	CCCGAGAGGAAGCAGGAATC
Fabp4	TGATGCCTTTGTGGGAACCT	TTGTGGTCGACTTTCCATCCC
PPARγ	GTGAGACCAACAGCCTGACG	CTTCCATCACGGAGAGGTCC
ACC	TGTACAAGCAGTGTGGGCTGGCT	CCACATGGCCTGGCTTGGAGGG
FAS	GAGGACACTCAAGTGGCTGA	GTGAGGTTGCTGTCGTCTGT
LPL	GCCTTTCTCCTGATGACGCT	AACTCAGGCAGAGCCCTTTC
ATGL	GACAGCTCCACCAACATCCA	GAGGCGGTAGAGATTGCGAA
PLIN	CCCGGCTCTTCAATACCCTC	TGGTGGCAGGAGGAACTCTA
Srebp-1c	GGAGCCATGGATTGCACATT	GGCCCGGGAAGTCACTGT
Fatp1	CGCTTTCTGCGTATCGTCTGCAAG	AAGATGCACGGGATCGTGTCT
Fatp2	CTGATGATCGACCGTGAGAA	TACCAGTCCCACGATGTCAG
Fatp3	AGGCTGCTCGAATCAGTCAT	AACTTGGGTTTCAGCACCAC
Fatp4	CAGCAACTGTGACCTGGAGA	CCTTCCGCAACTCTGTCTTC
Fatp5	GGTTTTTGCATTCCTGTGGA	GAAGGGTTGGTTCTTTCGAA
Cd36	GGCAACCAACCACAAATTAGCA	AAGGCTAGGAAACCATCCACC
CPT-1α	TTGCACGAGGGAAAAATAAGC	CCCTGCATGCGGTGGAAAAGGC
F4/80	TGGAATGTCAAGTCTGCACCA	GTGGCAGGTTGCATGTTCAG
Cd68	GGGGCTCTTGGGAACTACAC	GCCATGAATGTCCACTGTGC
MCP1	AGCTGTAGTTTTTGTCACCAAGC	TGCTTGAGGTGGTTGTGGAA
IL-1β	AATGCCACCTTTTGACAGTGATG	ATGTGCTGCTGCGAGATTTG

TNF-α	AGGCACTCCCCCAAAAGATG	CCACTTGGTGGTTTGTGAGTG
IFN-γ	GGGTTGTATCTGGGGGTGGG	GTCACTGCAGCTCTGAATGTTTCTT
iNOS	CCCAGTTGTGCATCGACCTA	ACCACTCGTACTTGGGATGC
β-actin	ACACTGTGCCCATCTACGAG	CAGCACTGTGTTGGCATAGAG

Supplementary Table 1. The list of primers sequence.

Genes	ARE sites	Function about adipose tissue
Isl1	6	regulating insulin gene expression；associated with maturity-onset diabetes of the young
Ces1f	2	hydrolysis of short-chain fatty acid ester；promote adipocyte lipolysis
Fgf1	1	a metabolic hormone crucial for the management of nutrient stress, glycaemic control and insulin sensitivity
Cdh11	21	regulate adipose tissue inflammation
Ctf1	8	common genetic variation in CTF1 could contribute to insulin sensitivity in humans
Pon1	1	potent TAG-lowering property
Nnt	2	NNT mRNA expression is significantly higher in visceral fat of obese patients and correlates with body weight, BMI, % body fat, visceral and sc fat area, waist and hip circumference, and fasting plasma insulin
Acsm3	7	
Pcdhgb1	2	
Pcdhgb4	2	
Tnfrsf11b	12	
Mmp9	5	
Gjb3	1	
Hcar2	1	
Tmem255a	8	

Myrf	2
Fabp3	1
Wt1	2
Lrrn4	1
Stmn2	4
Tmem151a	3
Wnt2b	6
Upk3b	1
Ppl	1
Zbtb7c	2
Pfkfb3	8
Lamc3	6
Osr1	3
Atp1b1	12
Nkx2-9	3
Efs	3
Cdc42se1	4
Cd24a	4
Tnk1	1
Adgrd1	2
Wfikkn2	1
Il22ra1	1

2010300C02Rik	1
Capn6	5
Rtn1	1
Scn3b	2
Stk26	15
Fam84a	5
Emp2	2
Crb2	1
Dkk3	4
Gpcpd1	5
Aldh1a2	6
Ildr2	13
Ugt1a1	4
Bnc1	10
Hmgn2	1
Flvcr2	5
Lrrc1	5
Bco2	1
Faim2	1
Rasl10b	2
Matn4	1
Sv2a	3

Thbd	8
Bhlhe41	12
Arsg	1
Baiap2l1	8
Csrp2	1
Cldn10	2
Nav3	1
Lrrc4	7
Ccdc122	1
Gata6	6
Smtnl2	4
Ikzf2	34
Cgnl1	4
1700029J07Rik	1
Traf3ip2	2
Slc23a4	1

Supplementary Table 2. The list of potential HuR targets.
