# Supplementary material for: Adipose HuR protects against diet-induced obesity and insulin resistance
Source: Nat Commun. 2019 May 30;10:2375. doi: 10.1038/s41467-019-10348-0 (PMC6542850; doi:10.1038/s41467-019-10348-0)
Supplement: Supplementary file 3 — Description of Additional Supplementary Files [file 41467_2019_10348_MOESM3_ESM.pdf]

Description of Additional Supplementary Files

File name: Supplementary Data 1

Description: The differentially expressed genes in HuR-deficient adipose tissues (>1.5 fold change). #165,196,210: Control; #162,193,227: HuR^{AKO}.
