# Supplementary material for: Author Correction: Adipose HuR protects against diet-induced obesity and insulin resistance
Source: Nat Commun. 2026 Nov 4;17:11415. doi: 10.1038/s41467-026-79109-0 (PMC13737080; doi:10.1038/s41467-026-79109-0)
Supplement: Supplementary file 1 [file 41467_2026_79109_MOESM1_ESM.pdf]

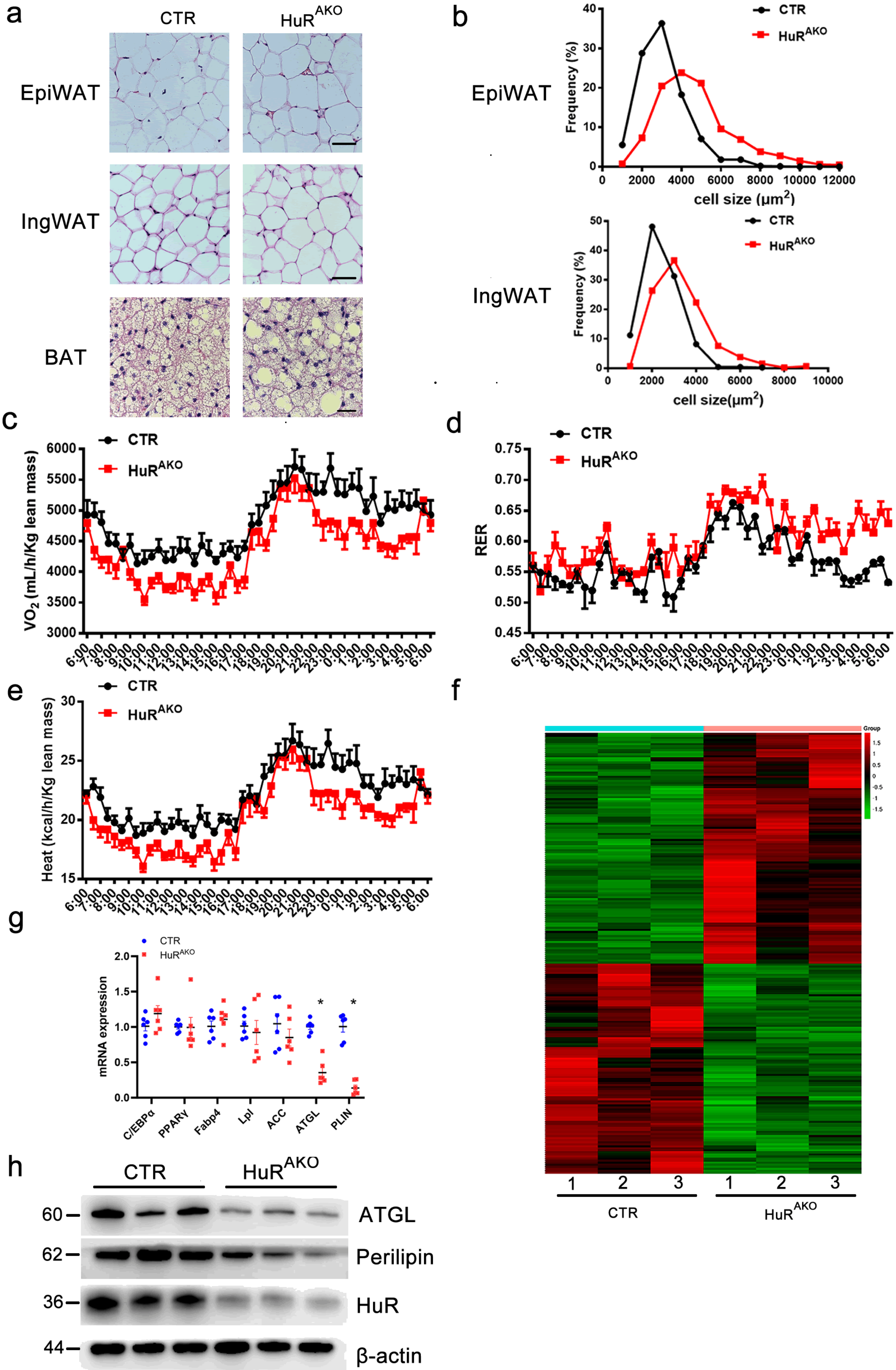

a
CTR
HuR^AKO
EpiWAT
IngWAT
BAT
b
EpiWAT
Frequency (%)
cell size (μm²)
CTR
HuR^AKO
IngWAT
Frequency (%)
cell size(μm²)
c
CTR
HuR^AKO
VO2 (mL/h/Kg lean mass)
d
CTR
HuR^AKO
RER
e
CTR
HuR^AKO
Heat (kcal/h/Kg lean mass)
f
Group
1 2 3
CTR
1 2 3
HuR^AKO
g
CTR
HuR^AKO
mRNA expression
C/EBPα
PPARγ
Fabp4
Lpl
ACC
ATGL
PLIN
h
CTR
HuR^AKO
60
ATGL
62
Perilipin
36
HuR
44
β-actin

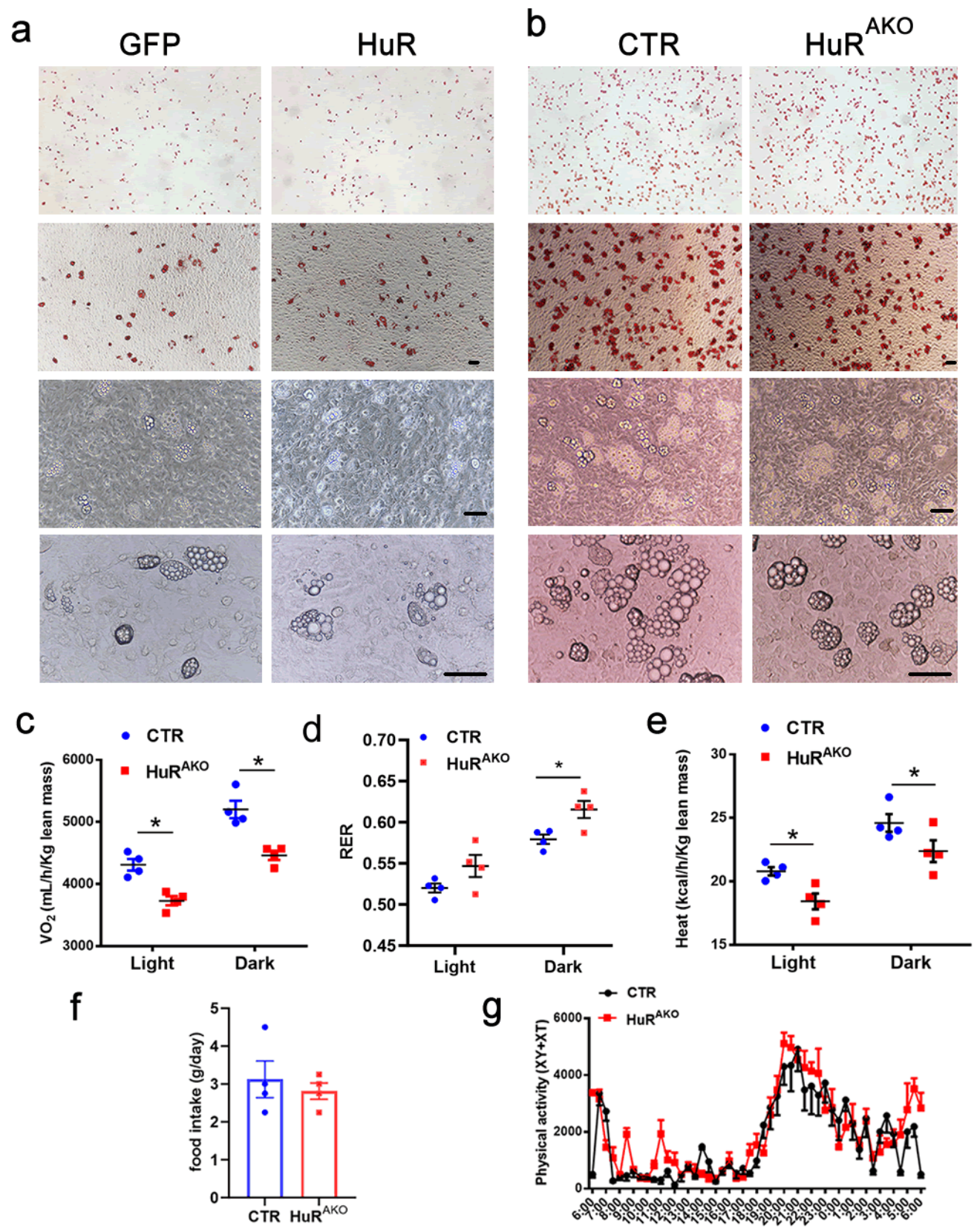
a
GFP
HuR
b
CTR
HuRAKO
c
CTR
HuRAKO
VO2 (mL/h/Kg lean mass)
6000
5000
4000
3000
*
*
Light
Dark
d
CTR
HuRAKO
RER
0.70
0.65
0.60
0.55
0.50
0.45
*
Light
Dark
e
CTR
HuRAKO
Heat (kcal/h/Kg lean mass)
30
25
20
15
*
*
Light
Dark
f
food intake (g/day)
5
4
3
2
1
0
CTR
HuRAKO
g
CTR
HuRAKO
Physical activity (XY+XT)
6000
4000
2000
0
